# Possible Involvement of Endothelin Peptides and L-Arginine-Nitric Oxide Pathway on the Effect of Endotoxin in the Rabbit Isolated Perfused Kidney

**DOI:** 10.1155/S0962935194000293

**Published:** 1994

**Authors:** K. Özsan, R. K. Türker, Z. S. Ercan

**Affiliations:** 1 Department of Microbiology Faculty of Medicine University of Ankara Turkey; 2 Department of Pharmacology Faculty of Medicine University of Ankara Turkey; 3 Department of Pharmacology Faculty of Medicine Gazi University Ankara Turkey

## Abstract

*Escherichia coli* endotoxin (LPS) when infused through the renal artery of the rabbit isolated perfused kidney prepared as constant pressure mode, caused a decrease in flow rate and kidney weight indicating its primary vasoconstrictor effect. This effect was predominant in kidneys from rabbits pretreated with LPS. Endothelin-1 at a concentration of 10^−10^ M and big endothelin-1 at a concentration of 10^−8^ M produced equal vasoconstrictor effects in kidney. Addition of endotheHn converting enzyme inhibitor, phosphoramidon, to the perfusion medium at a concentration of 10^−6^ M caused a reduction in the effects of both LPS and big ET-1 without altering the vasoconstrictor effect of ETol. However, addition of methylene blue (10^−5^ M), a soluble guanylate cyclase inhibitor and N^G^-nitro-L-arginine-methyl ester (10^−6^ M) to the perfusion medium caused a potentiation in the vasoconstrictor effect of LPS. Indomethacin at a concentration of 10^−6^ M did not alter the effect of LPS. These results were taken as evidence for the participation of endothelin peptides and the L-arginine-nitric oxide pathway in the effect ofLPS in rabbit isolated perfused kidney.


Mediators of Inflammation 3, 211–214 (1994)

### Possible involvement of endothelin peptides and L-arginine–nitric oxide pathway on the effect of endotoxin in the rabbit isolated perfused kidney

K. Özsan,[1] R. K. Türker[2,CA] and Z. S. Ercan[3]

Departments of [1]Microbiology and [2]Pharmacology, Faculty of Medicine, University of Ankara; [3]Department of Pharmacology, Faculty of Medicine, Gazi University, Ankara, Turkey

[CA] Corresponding Author

**ESCHERICHIA COLI endotoxin (LPS) when infused through the renal artery of the rabbit isolated perfused kidney prepared as constant pressure mode, caused a decrease in flow rate and kidney weight indicating its primary vasoconstrictor effect. This effect was predominant in kidneys from rabbits pretreated with LPS. Endothelin-1 at a concentration of $10^{-10}$ M and big endothelin-1 at a concentration of $10^{-8}$ M produced equal vasoconstrictor effects in kidney. Addition of endothelin converting enzyme inhibitor, phosphoramidon, to the perfusion medium at a concentration of $10^{-6}$ M caused a reduction in the effects of both LPS and big ET-1 without altering the vasoconstrictor effect of ET-1. However, addition of methylene blue ($10^{-5}$ M), a soluble guanylate cyclase inhibitor and $N^G$-nitro-L-arginine-methyl ester ($10^{-6}$ M) to the perfusion medium caused a potentiation in the vasoconstrictor effect of LPS. Indomethacin at a concentration of $10^{-6}$ M did not alter the effect of LPS. These results were taken as evidence for the participation of endothelin peptides and the L-arginine-nitric oxide pathway in the effect of LPS in rabbit isolated perfused kidney.**

**Key words:** *E. coli* LPS, Big ET-1, ET-1, Kidney, L-NAME, Methylene blue, Nitric oxide, Phosphoramidon

### Introduction

Bacterial endotoxin is known to cause damage in endothelial cells altering the structure, metabolism and permeability.[1] Peripheral vascular failure, in response to catecholamines and other vasoconstrictors, is the major factor causing death in human septic shock and endotoxin injected animals.[2,3] Recently $N^G$-nitro-L-arginine-methyl ester (L-NAME) an inhibitor of nitric oxide (NO) synthase, was shown to reduce hypotension induced by endotoxin (*Escherichia coli* lipopolysaccharide, LPS) and restore the pressor effects of noradrenaline (NA) and other vasoconstrictors.[4,5] These observations indicate that one of the mechanisms of vascular hyporeactivity induced by LPS is the increased production of NO from L-arginine (L-Arg) in blood vessels by activation of NO synthase. This hypothesis was supported by the study of Knowles *et al.*[6] who showed that LPS activates the production of NO in the rat aorta. Two distinct isoforms of NO synthase have been described. One is the constitutive $Ca^{2+}$/calmodulin dependent form which is mainly localized in endothelial cells; the other, however, is an inducible $Ca^{2+}$/calmodulin independent enzyme which predominates in vascular smooth muscle cells.[7]

Infusion of LPS was shown to increase blood pressure[8] associated with an increase of endothelin-1 (ET-1) levels in rat serum.[9] Furthermore, LPS can stimulate the release of ET-1 from cultured bovine aortic endothelial cells.[9] It has been shown that plasma and pulmonary lymph levels of ET-1 increase significantly during endotoxic shock.[10]

These results indicate that LPS causes the release or overproduction of endothelium derived vasoactive substances. The data presented in this paper give evidence of the release of NO and ET peptides by LPS in the rabbit isolated perfused kidney.

### Material and Methods

Experiments were performed on isolated perfused kidneys from adult rabbits of both sexes weighing 2.5–3.0 kg. Either LPS (5 mg/kg) or saline (1 ml as control) was injected i.p. and 4 h later rabbits were anaesthetized with sodium thiopental (20 mg/kg i.v.) and then premedicated with sodium heparin (500 IU/kg i.v.). Both kidneys were removed after cannulation of the renal arteries with appropriate polyethylene tubing as described previously.[11,12] The ureter was cut at its upper part near to the pelvis renalis to eliminate any resistance during urine discharge. The renal vein was also cut and venous return was discarded. Perfusion medium was Krebs–Hanseleit solution with the following composition: (mM) $Na^+$, 138.2; $K^+$, 5; $Ca^{2+}$, 2.5; $Mg^{2+}$, 0.5; $Cl^-$, 123; $HCO_3^-$, 25; $H_2PO_4^-$, 1.2; dextrose, 11.5; gassed with 95% $O_2$ + 5% $CO_2$ mixture, giving pH 7.4 at 37°C. Perfusion was provided in constant pressure mode at 80–100 mmH$_2$O. Flow changes were evaluated by means of the pressure drop at the T tube (Venturi

© 1994 Rapid Communications of Oxford Ltd

Tube) connection and correlated with the response of a pressure transducer (Statham P23 XL). Organ weight was also measured simultaneously throughout the experiment by means of a force displacement transducer (Grass FT.03). At a constant pressure mode (80–100 $mmH_2O$) the basal flow was measured and recorded as the maximal flow rate, which was estimated as 5.0–8.0 ml/min. Zero flow rate was recorded by intermission of circuit. Thus, changes in flow rate were evaluated at this range for individual experiments.

All chemicals used in this study were dissolved in saline. *E. coli* B-lipopolysaccharide endotoxin (lot number 055:35, Difco Lab., Detroit, MI, USA) was prepared at a concentration of 1% before use. Stock solutions of porcine ET-1 and big ET-1 (Sigma) ($10^{-5}$ M) were kept frozen at –20°C. Further dilutions were made in Krebs solution just before use and were infused through the renal artery at the same flow rate (0.1 ml/min). A stock solution ($10^{-2}$ M) of Phos (Sigma), an endothelin converting enzyme inhibitor,[13,14] was also kept frozen at –20°C. Further dilution was made in Krebs buffer. Methylene blue (MeB), a soluble guanylate cyclase inhibitor[15] and L-NAME (Sigma), a NO synthase inhibitor,[4,5] were freshly prepared in saline and added to the incubation buffer for individual experiments. Indomethacin (IND, Sigma) was dissolved in absolute ethanol and further dilutions were made in saline just before use. Following the control measurements of LPS and ET peptides, Phos ($10^{-6}$ M), MeB ($10^{-5}$ M), L-NAME ($10^{-6}$ M) and IND ($10^{-6}$ M) were added to the incubation medium and kidneys were allowed to perfuse for at least 20–30 min. The tests were repeated in a separate series of experiments.

All data were expressed as the arithmetic mean ± S.E.M. The statistical significance of differences from the control was estimated using paired Student's *t*-test, *p* values less than 0.05 were considered significant.

### Results

The maximal flow rate of Krebs perfused kidneys with constant pressure mode (80 $mmH_2O$) from LPS pretreated rabbits was calculated as 8.7 ± 0.9 ml/min in a series of experiments ($n = 15$). Infusion of LPS into the renal artery for 2 min (total 100 μg) produced a decrease in flow and kidney weight indicating its predominant vasoconstrictor effect as shown in the first parts of Figs 1 and 2. The response in both parameters reached the maximal level within 4.0 ± 1.0 ($n = 15$) min in kidneys from LPS pretreated rabbits but more than 20 min in kidneys from intact animals. Addition of MeB to the incubation medium at the concentration of $10^{-5}$ M caused a potentiation in both parameters as shown in the second part of Fig. 1. Similar potentiated responses in both parameters against LPS were obtained when kidneys were pretreated with L-NAME. The calculated results of a series of experiments are shown in Table 1, indicating a highly significant difference ($p < 0.01$) in both parameters when compared with their corresponding controls. In another series of experiments,

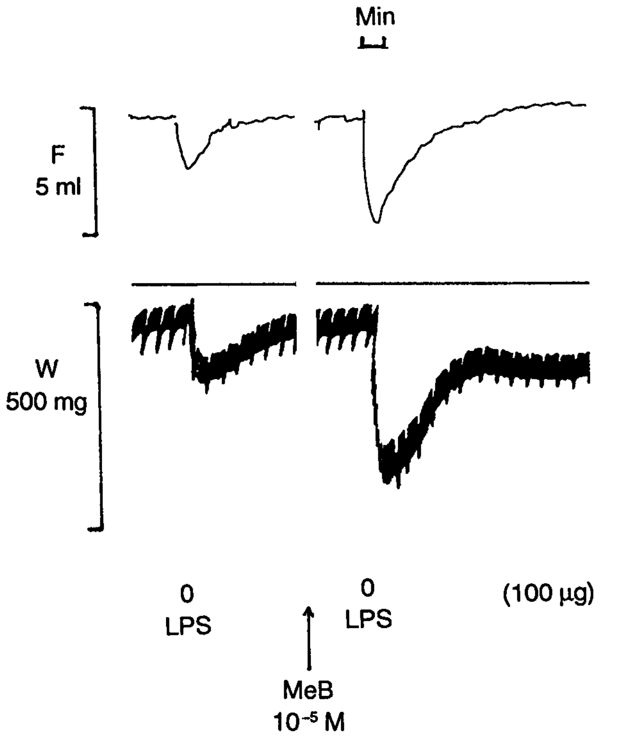

FIG. 1. A typical recorder tracing of the isolated perfused kidney from LPS pretreated rabbit showing the effect of LPS infusion on flow rate (F) and kidney weight (W) before and after addition of methylene blue (MeB) into the perfusion medium. Response to LPS increased significantly after MeB.

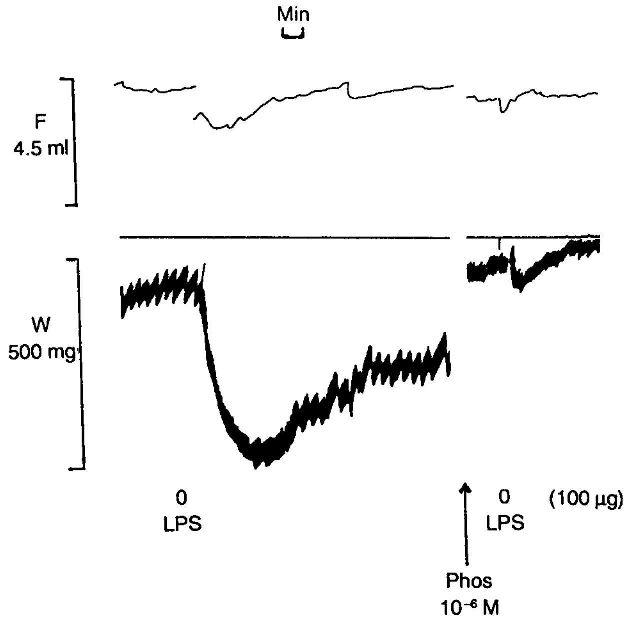

FIG. 2. A recorder tracing of the isolated perfused kidney with constant pressure mode from LPS treated rabbit representing the effect of LPS infusion for 2 min before and after phosphoramidon (Phos). Upper part indicates flow rate (F), lower part shows kidney weight (W). Response to LPS infusion for 2 min (total amount 100 μg) markedly decreased after Phos.

**Table 1.** Decrease in flow rate and kidney weight by LPS and changes induced by MeB, L-NAME and Phos

| Compound added | Decrease in flow rate (Δ ml) | Number of experiments (*n*) | Decrease in kidney weight (Δ mg) |
|---|---|---|---|
| Control LPS | 1.83 ± 0.25 | 15 | 310.0 ± 22.0* |
| MeB + LPS | 3.40 ± 0.40 | 15 | 418.0 ± 32.0** |
| Control LPS | 2.45 ± 0.70 | 15 | 375.0 ± 34.0* |
| Control LPS | 1.76 ± 0.93 | 7 | 305.0 ± 43.2* |
| L-NAME + LPS | 4.71 ± 0.73 | 7 | 395.0 ± 53.6** |
| Phos + LPS | 0.75 ± 0.12 | 15 | 93.0 ± 11.5** |

*,** $p < 0.01$ when compared with their corresponding controls.
Amount of each compound used was: LPS, 100 mg; MeB, $10^{-5}$ M; L-NAME, $10^{-6}$ M; Phos, $10^{-6}$ M.
Values are the mean ± S.E.M.

**Table 2.** Decrease in flow rate and kidney weight by ET-1 and big ET-1 and changes induced by Phos

| Compound added | Decrease in flow rate (Δ ml) | Decrease in kidney weight (Δ mg) |
|---|---|---|
| ET-1 | 5.80 ± 1.3 | 420.0 ± 41.5 |
| Big ET-1 | 4.67 ± 2.3 | 388.0 ± 28.6* |
| Phos + ET-1 | 6.70 ± 2.6 | 440.0 ± 55.0 |
| Phos + big ET-1 | 1.70 ± 0.9 | 120.0 ± 21.6** |

*,** $p < 0.01$ when compared with their corresponding controls.
Amount of each compound used was: ET-1, $10^{-10}$ M; big ET-1, $10^{-8}$ M; Phos $10^{-6}$ M.
Values are the mean ± S.E.M.

Phos, added to the perfusion medium at the concentration of $10^{-6}$ M, inhibited the effects of LPS significantly ($p < 0.01$) in perfusion flow and kidney weight as shown in Fig. 2 (second part) and Table 1. Phos also reduced the effect of big ET-1 without altering the effect of ET-1 in both parameters. The calculated results are shown in Table 2. No appreciable change was observed in both parameters against LPS and ET-1 when the experiments were repeated in the presence of IND ($10^{-6}$ M).

### Discussion

The results of this study indicate that both NO and ET peptides play an important role in the mediation of responses caused by LPS in the rabbit isolated perfused kidney. Changes in renal flow and organ weight under constant pressure mode of perfusion system are two sensitive and reproducible parameters for the evaluation of vasoactive substances in isolated perfused kidney. According to the results of the present study LPS caused a decrease in renal flow and kidney weight, indicating its main vasoconstrictor effect when infused into the renal artery of the rabbit isolated perfused kidney. Other vasoconstrictors such as noradrenaline and angiotensin II also elicit a decrease in flow rate and kidney weight when injected into the renal artery (data not shown). ET-1 and big ET-1 caused similar vasoconstrictor response as measured by both parameters when directly infused into the renal artery. The vasoconstrictor effect of LPS attenuated significantly after the treatment of kidney with Phos, a known metallopeptidase inhibitor which inhibits the conversion of big ET-1 to ET-1[16] indicating the primary role of ET peptides in the mechanism of LPS induced vasoconstriction. It is well known that endothelial cells of the resistance vessels of kidney produce ET peptides.[17] The vasoconstrictor response to LPS is a rapid phenomenon in the kidneys from LPS pretreated animals when compared with the response observed in untreated kidneys, indicating a predisposing and promoting action of LPS on the cascade production of ET peptides in kidney.

The results of the present study also indicate that the production of NO in resistance vessels of kidney can contribute to the effect of LPS. The effect of LPS is prominent on kidney vasculature in LPS treated animals, supporting the results of Schneider *et al.*[18] who showed an elevation of L-arg derived NO in small mesenteric arteries from endotoxin treated rats. It has been reported that LPS induced NO synthase activity in cultured endothelial cells[19] and endogenous L-arg is sufficient for maximal NO production by the calmodulin activated constitutive NO synthase in endothelial cells.[20] It has been shown previously that acetylcholine produces vasodilatation in the isolated perfused rabbit kidney when submaximal vasoconstriction is elicited by phenylephrine.[12] Removal of endothelium by Triton X-100[21] or pretreatment of kidney with MeB, a soluble guanylate cyclase inhibitor,[15] completely abolished the vasodilator effect of acetylcholine, indicating that this response is mediated by the release of EDRF (NO) from the resistance vessels of kidney.[12] In the present study, prior treatment of kidney with MeB greatly enhanced the vasoconstrictor effect of LPS as evidenced by the significant decrease of flow rate and kidney weight. Specific NO synthase inhibitor L-NAME also produced similar potentiation as observed with MeB indicating the role of L-arg–NO pathway on the effect of LPS in rabbit isolated perfused kidney. IND did not alter the effect of LPS in kidney, indicating lack of participation of prostanoids in rabbit isolated perfused kidney.

All these results strongly indicate that LPS causes an increase in the production of both NO and ET peptides in the isolated perfused kidney from rabbits pretreated with LPS. These results also indicate that in various pathological conditions elicited by endotoxin, the L-arg–NO pathway and ET peptides are important endogenously occurring mediators.

### References

1. Meyrick BO, Ryan US, Brigham KL. Direct effects of *E. coli* endotoxin on structure and permeability of pulmonary endothelial monolayers and the endothelial layer of initial explant. *Am J Pathol* 1986; **122**: 140–151.

2. Parratt JR. Alterations in vascular reactivity in sepsis and endotoxemia. In: Vincent JL, ed. *Update in Intensive Care and Emergency Medicine No. 5.* Berlin: Springer-Verlag, 1987; 299–312.
3. Güc, MO, Furman BL, Parratt JR. Modifications of α-adrenoceptor-mediated pressor responses by $N^G$-nitro-L-arginine methyl ester and vasopressin in endotoxin-treated pithed rats. *Eur J Pharmacol* 1992; **224**: 63–69.
4. Thiemermann C, Vane JR. Inhibition of nitric oxide synthesis reduces the hypotension induced by bacterial lipopolysaccharides in the rat *in vivo*. *Eur J Pharmacol* 1990; **182**: 591–595.
5. Gray GA, Schott C, Julo-Schaeffer G, Fleming I, Parratt JR. The effect of inhibitors of the L-arginine/nitric oxide pathway on endotoxin-induced loss of vascular responsiveness in anaesthetized rats. *Br J Pharmacol* 1991; **103**: 1218–1224.
6. Knowles RC, Salter M, Brooks SL, Moncada S. Antiinflammatory glucocorticoids inhibit the induction by endotoxin of nitric oxide synthase in the lung, liver and aorta of the rat. *Biochem Biophys Res Commun* 1990; **172**: 1042–1048.
7. Moncada S, Palmer RMJ, Higgs EA. Nitric oxide: physiology, pathophysiology and pharmacology. *Pharmacol Rev* 1991; **43**: 109–142.
8. Kikeri D, Pennel JD, Hwang KH, Jacob AI, Richman AV, Bourgolgnic JJ. Endotoxemic acute renal failure in awake rats. *Am J Physiol* 1986; **250**: F1098–F1106.
9. Sugiura M, Inagami T, Kon V. Endotoxin stimulates endothelin-release *in vivo* and *in vitro* as determined by radioimmunoassay. *Biochem Biophys Res Commun* 1989; **161**: 1220–1227.
10. Morel KR, Lacroix JS, Hemsen A, Steinig DA, Pittet J-F, Lundberg JM. Increased plasma and pulmonary lymph levels of endothelin during endotoxin shock. *Eur J Pharmacol* 1989; **167**: 427–428.
11. Türker RK, Demirel E, Ercan ZS. Iloprost preserves kidney functions against anoxia. *Proc Leuk Essen Fatty Acids* 1988; **31**: 45–52.
12. Ercan ZS, Soydan AS, Türker RK. Possible involvement of endothelium in the responses of various vasoactive agents in rabbit isolated perfused kidney. *Gen Pharmacol* 1990; **21**: 205–209.
13. Okada K, Miyazaki Y, Takada J, Matsuyama K, Yamaki T, Yano M. Conversion of big endothelin-1 to endothelin-1 by membrane bound metalloproteinase in cultured bovine endothelial cells. *Biochem Biophys Res Commun* 1990; **171**: 1192–1198.
14. Matsumura Y, Ikegawa R, Tsukahara Y, Takaoka M, Moriomoto S. Conversion of big endothelin-1 to endothelin-1 by two types of metalloproteinases of cultured porcine vascular smooth muscle cells. *Biochem Biophys Res Commun* 1991; **178**: 899–905.
15. Martin W, Villani GM, Jothinandan D, Furchgott RF. Selective blockade of endothelium-dependent and glyceryl trinitrate induced relaxation by hemoglobin and methylene blue in rabbit aorta. *J Pharmacol Exp Ther* 1985; **232**: 708–716.
16. Ikegawa R, Matsumura Y, Tsukahara Y, Takaoka M, Morimoto S. Phosphoramidon, a metalloproteinase inhibitor, suppresses the secretion of endothelin-1 from cultured endothelial cells by inhibiting a big endothelin-1 converting enzyme. *Biochem Biophys Res Commun* 1990; **171**: 669–675.
17. Rubanyi GM, Parker Botelho LH. Endothelins. *FASEB J* 1991; **5**: 2713–2720.
18. Schneider F, Jean-Claude Stoclet CS, Julo-Schaeffer G. L-Arginine induces relaxation of small mesenteric arteries from endotoxin-treated rats. *Eur J Pharmacol* 1992; **211**: 269–272.
19. Radomski MW, Palmer RMJ, Moncada S. Glucocorticoids inhibit the expression of an inducible, but not the constitutive, nitric oxide synthase in vascular endothelial cells. *Proc Natl Acad Sci* 1990; **87**: 10043–10047.
20. Mitchell JA, Hecker M, Anggard EE, Vane JR. Cultured endothelial cells maintain their L-arginine level despite the continuous release of EDRF. *Eur J Pharmacol* 1990; **182**: 573–576.
21. Connor HE, Feniuk W. Role of endothelium in haemoglobin-induced contraction of dog basilar artery. *Eur J Pharmacol* 1987; **140**: 105–108.

ACKNOWLEDGEMENT. This work was supported by a grant from the Turkish Scientific and Technical Research Council (TAG-0745).

**Received 10 February 1994; accepted in revised form 11 March 1994**
